# As Plain as the Nose on Your Face: The Case for A Nasal (Mucosal) Route of Vaccine Administration for Covid-19 Disease Prevention

**DOI:** 10.3389/fimmu.2020.591897

**Published:** 2020-09-30

**Authors:** Craig R. Travis

**Affiliations:** Immugen Pharma LLC, South Miami, FL, United States

**Keywords:** vaccine, mucosal, IgA, IgG, antibody-dependent enhancement, Covid-19, intramuscular

At present, the target of most of the SARS-CoV-2 (Covid-19) vaccine development worldwide is the spike protein (S) or, more specifically, the receptor-binding domain (RBD) of the virus (1). According to The World Health Organization, almost all of these vaccines will be delivered parentally by intramuscular injection (2). The goal is to achieve broadly neutralizing IgG antibody production in response to a systemic viremia and contribute to the mucosal immune defense. However, questions remain about the relative impact that IgG makes to the mucosal response, whether or not it can provide durable immunity, especially in the aging population, and to what degree it contributes to the immunopathology of antibody-dependent enhancement (ADE).

Despite the reliance on the intramuscular approach, mucosal administration of vaccines has been highly successful from ancient through modern times (3). The late Norwegian immunologist, Per Brandtzaeg, was a strong advocate for the intranasal administration of vaccines because of the regional effect that it has on the upper airways with the production of both systemic and mucosal IgA and systemic IgG immunoglobulins (4). He was also highly critical of the surgical removal of the adenoids and tonsils in children, in part, due to impaired responses to vaccines (5). Perhaps the pediatric population is being spared the ravages of the current pandemic due to the protective nature of the adenoids and tonsils.

The tonsils and adenoids are part of the mucosal immune system known as Waldeyer’s ring or the nasal associated lymphoid tissue (NALT). This organized mucosal associated lymphatic tissue lies below the lamina propria of the nasal mucosa and is the primary inductive site for the secretory immune system (6). It is in this region where all the molecular and cellular conditions are available for the production of secretory IgA (S-IgA) by plasma cells and memory-type IgA+ B cells independently of the bone marrow (7).

Plasma B cells produce both monomeric (sIgA) and polymeric (pIgA) multimers, dimers, tetramers and pentamers (8). This multivalency results in greater avidity for viral peptides than IgG (9) and prevents the infiltration of pathogens known as immune exclusion (10). The pIgA is actively transported across the cell membrane from the basolateral to the apical surface of the secretory epithelium by the secretory component (SC) of polymeric-immunoglobulin receptor (pIgR) as a secretory (SIgA) complex. As the SIgA reaches the surface of the uninfected cell, SC separates from the SIgA where both elements diffuse into the mucus layer and provide specific protective mechanisms (11). In vitro, free SC binds to IL-8 and inhibits IL-8-mediated recruitment of neutrophils to prevent neutrophil extracellular traps in the airways (see below) (12, 13). If a cell has become infected by a virus, pIgA complex is absorbed through the basal membrane by the pIgR where it is then internalized into the endoplasmic reticulum leading to the intracellular neutralization of newly formed viral proteins which are then eliminated through the apical surface into the intestinal or airway lumen (14).

The expression of the peripheral node addressin (PNAd) by the high-walled endothelial venules of the NALT accounts for the trafficking of B and T lymphocytes to the salivary, parotid and submaxillary gland lymph nodes (15, 16) where plasma B cells then migrate to the salivary and parotid glands to express IgA that offers protection against bacterial pathogens produced in the oral cavity as well as inhaled airborne virions (17). The PNAd derived from NALT also promotes a mucosal and systemic humoral response that includes that includes the lungs (18) and the genital mucosa (19).

Given that PNAd is expressed by the HEV in the NALT and bronchial associated lymphatic tissues, its role in cellular immunity in response to vaccination is paramount since up to 80% of lymphocytes in human tonsils are CD8^+^ memory cells (20). On the other hand, naïve T cells were excluded from the mucosal-associated tissue in mice that were challenged with influenza virus that suggested a mechanism of immune tolerance in the upper airway. The activation of CD8^+^ cells by intranasal boosting with a recombinant vaccinia virus encoding the spike protein of the SARS-CoV in mice resulted in pathogen clearance from a lethal challenge of the virus (21). However, in Covid-19 patients, lymphopenia is the hallmark of disease progression (22) and in particular, CD8^+^ and natural killer cells (NK) decreased with progression of the disease (23). Not only does the innate immune response fail to protect against Covid-19, but it may be the underlying cause of the increased morbidity and mortality (24).

A large body of literature has demonstrated that protection of the lungs is afforded by nasal administration of a variety of viral and bacterial vaccines (25–27). While there are concerns about the durability of IgG antibodies to Covid-19 (28), IgA antibodies to influenza generated by the diffuse NALT lining the nasal passages lasted for the life of the animal (29).

The Covid-19 infection epitomizes a mucosal disease process. Close contact, aerosol droplets, and fomites facilitate the transmission of the virus where it comes into contact with the oronasal and conjunctival mucosa. Here, the spike protein of the virus binds to the angiotensin-converting enzyme-2 (ACE2) receptor of the target cells capable of replicating the virus (30). The nasal epithelium has the highest concentration of ACE2, and the alveoli have the lowest (31). These findings reflect that the most robust replication of the virus likely takes place in the nose and little or none in the alveoli (32). Furthermore, the epithelial cells lining the salivary gland ducts that are rich in the expression of ACE2 actively produce virions (33) that are spread through aerosol droplets (34) that may be inhaled or aspirated into the lung. (31, 35) However, since IgA seroconversion occurs two days after the onset of infection, and is detected earlier than IgM or IgG in Covid-19 patients (36), its presence in the saliva not only provides the basis for point-of-care diagnostic testing (37) but further supports the use of the intranasal administration of a vaccine in order to neutralize the virus at its source—the upper airway.

However, despite the presence of antigen presenting cells in the nasal and oral mucosa, SARS-CoV-1 was able to evade this innate immune response in Rhesus macaques (RM), and within two days post infection (dpi) had breached the upper respiratory epithelium where it infected the underlying mucosal dendritic cells and macrophages that subsequently migrated from Waldeyer’s ring to draining lymph nodes and into the lungs where they formed dense clusters around the alveoli (38). The sequestration of virus in intracellular vesicles of the macrophages demonstrates the critical role that these antigen presenting cells (APC) play in the dissemination of the virus to the lung and systemic compartment especially since viral shedding of Covid-19 in the pharynx precedes viral replication in the lungs (39).

While pre-existing immunity is considered beneficial, there is great concern that the accelerated pace to develop a vaccine against SARS-CoV-2 will result in a detrimental immune response, i.e., an antibody-dependent enhancement (ADE) of the infection (40). Particularly disturbing is the fact that as a result of prior exposure to the “common cold coronavirus” (CCC), T cell reactivity to SARS-CoV-2 antigen peptide pools is in the 20–50% range in unexposed blood donors from across the globe (41). In fact, one study showed that 90% of the human race tested positive for three of the four CCCs (42). A recent study showed that 35% of seronegative Covid-19 healthy donors had cross-reactive CD4+ T cells to the S protein probably acquired from previous infections with human coronaviruses (43). The presence of durable cross-reactive T cell memory responses would play a role in amplifying an anamnestic B cell response against those common antigens (44, 45). Thus, prior sensitization to conserved epitopes could lead to the production of non- neutralizing or sub-neutralizing binding antibodies, principally of the IgG isotype, and form antigen-antibody complexes. These immune complexes (IC) act as molecular bridges between a virus and immune cells (46) expressing either a complement receptor, IgG Fc receptor (FcγR) on the surface and neonatal Fc receptor (FcRn) (47) intracellularly. The FcγR can function as a mimic for the ACE2 receptor that is not expressed on all immune cells and allows for neutralizing antibodies to gain access to the reproductive machinery of those cells (48). Ultimately, the ratio of activating versus inhibitory FcγRs will determine the severity of the disease based on the subtype of IgG that it binds and the subsequent signaling cascades it produces (49). When the IC binds to an activating FcγR on APCs it also results in the production of proinflammatory cytokines and chemokines that lead to lung and other organ injury (50, 51). This hypercytokinemia causes an increased transudate and production of hyaluronan in the alveoli that absorbs up to 1,000 times its molecular weight with water resulting in the severe acute respiratory syndrome (SARS) and death (52). Even though ADE is primarily associated with IgG antibodies, the phenomenon has also been observed with IgA antibodies in HIV infection (53, 54). However, other than HIV, IgA has not been identified with ADE in any other viral infection.

Two recent studies confirmed that fully neutralizing IgG antibodies led to disease enhancement. One study showed that monoclonal antibodies targeting the MERS-CoV RBD caused a conformational change in the spike protein that blocked viral entry into cells expressing its cognate receptor, dipeptidyl peptidase 4 and directed its entry into FcγR expressing cells (55). In the second study, an IM vaccination that produced an anti-spike IgG (S-IgG) and an intravenous administration of S-IgG monoclonal antibodies correlated with acute lung injury during a SARS-CoV infection of RM (56). Although the anti-S-IgG reduced the expression of viral RNA in the lungs, it led to a massive accumulation of monocyte/macrophages within 2 dpi that caused significant diffuse alveolar damage. An antibody directed against the FcγR reduced the production of IL-8 and MCP1 by wound-healing macrophages suggesting that the mechanism of acute lung injury was mediated by the anti-S-IgG antibody.

It is not known if the expression of neonatal Fc receptor (FcRn) in the endothelial, airway and gastrointestinal tissues (57) plays a role in IgG mediated enhancement of SARS-CoV-2. Coronaviruses as well as other viruses that form immune complexes with IgG antibodies are transcytosed through the plasma membrane and transported intracellularly by the FcRn into the endosomal system (58). Both the IgG antibody (59) and the mouse hepatitis virus, a prototypic member of the CoV family (60), depend on the same Rab GTPases in the endosomal system for the recycling of IgG and for the proteolytic processing of their fusion proteins respectively. This escorted means of endocytosis of the virus could be the underlying mechanism of the endovascular events observed late in the infection (61). Of particular note, 82% of the cases of Kawasaki-like disease in children in France had IgG antibodies for SARS-CoV-2 (62).

Although the induction of a mucosal response by systemic immunization remains poorly understood (63), the use of an appropriate adjuvant could change the outcome and lead to the expression of IgA (64). Nevertheless, the intranasal administration of a vaccine is inherently associated with an IgA response. An additional benefit of IgA is based on its non-inflammatory effects since neither the secreted, monomeric form (sIgA) found in serum nor the secretory, polymeric form (S-IgA) found in mucosal secretions activate any of the three complement pathways (65, 66). And, when bound to the antigen, IgA blocks the binding of IgG and IgM and thus prevents the complement-mediated inflammatory effects associated with these isotypes (67). Furthermore, all forms of the IgA antibody, serum and secretory, monoclonal and polyclonal, interfered with complement-dependent phagocytosis by neutrophils mediated by IgG antibodies (66). This would be beneficial in limiting the recruitment of neutrophils to the lungs and the inflammasomes associated with viral infections (68).

In the context of a coronavirus vaccine, two separate studies compared the efficacy of an intramuscular versus a mucosal route. The first study used a recombinant adeno-associated virus (rAAV)-based RBD (RBD-rAAV) vaccine to the SARS-CoV spike protein (69) and the second studied three adenovirus-based vaccine candidates against MERS-CoV (70). In both studies, the intranasal route was superior to the IM route in terms of a systemic and local humoral response, and both had a stronger systemic and pulmonary CTL response. However, neither the IM nor intranasal administration of the SARS-CoV RBD-rAAV vaccine produced any ADE which the authors attributed to the properties of the adenovirus vector and its specificity for the particular epitope within the RBD. But perhaps most importantly, only the intranasal and subligual administration of the MERS-CoV full-length spike protein induced IgA antibodies that were found in the broncholaveolar lavage fluid. Thus far, only one paper has clearly substantiated the validity of previous articles that support the nasal administration of a Covid-19 vaccine (71) although a number of academic and biopharma entities have announced their successes with press releases.

While IgA is the most highly expressed antibody in the body, its production by the mucosal-associated lymphatic tissue declines with age. This decline is one aspect of a condition known as immunosenescence that is particularly relevant in the current pandemic caused by Covid-19 in which the elderly are the most vulnerable population. However, a study in mice showed that the aging process affects the NALT to a lesser degree than the gastrointestinal associated lymphatic tissue (72). This suggests that all of the necessary immunocompetent cells are maintained in the nasal mucosa to mount an effective immune response. However, the need still remains to determine an appropriate adjuvant for mucosal administration (73) of a Covid-19 vaccine especially one that would avoid a Th17 response that contributes to the eosinophilic infiltration in the lungs (74).

If seen only from an immunological perspective, the IM administration of a vaccine is not without its drawbacks (75). There is a significant concern about the lack of availability of vials, needles, and syringes to meet the global demand. Then, there is the need for trained personnel to administer the vaccine intramuscularly that can result in as many as five needle-stick injuries per 100 injections worldwide (76). Also, there may be poor compliance due to the anticipated pain at the injection site and concerns about the arms race mentality that may have flattened the traditional trajectory required for a safe and effective vaccine. And, lastly, there is a significant concern for the reuse of needles and syringes in developing countries that can lead to blood-borne viral infections and for the proper disposal of this medical waste in these countries.

Regulatory agencies worldwide should require a comparison of the parenteral administration with mucosal delivery and accelerate the approval of the appropriate adjuvants, particularly for the aging population. If successful, mucosal delivery will play a protective role in preventing the invasion of the virus early in the infectious process and prevent the viremia to which an IgG response is also generated. Mucosal delivery also represents a more cost-effective and efficient means of delivering a vaccine in the time of a pandemic. And ultimately, there is less likelihood of an immunopathological immune response known as ADE that is invariably associated with IgG.

## Author Contributions

The author confirms being the sole contributor of this work and has approved it for publication.

## Conflict of Interest

CT is the Founder and President of Immugen Pharma LLC.

## References

[1] Premkumar L Segovia-Chumbez B Jadi R. Martinez DR Raut R Markmann A The receptor binding domain of the viral spike protein is an immunodominant and highly specific target of antibodies in SARS-CoV-2 patients. Sci Immunol (2020) 5(48):eabc8413. 10.1126/sciimmunol.abc8413 32527802 PMC7292505

[2] Available at: https://www.who.int/publications/m/item/draft-landscape-of-covid-19-candidate-vaccines.

[3] Hellfritzsch M Scherließ R Mucosal Vaccination via the Respiratory Tract. Pharmaceutics (2019) 11(8):375. 10.3390/pharmaceutics11080375\ PMC6723941 31374959

[4] Brandtzaeg P Potential of nasopharynx-associated lymphoid tissue for vaccine responses in the airways. Am J Respir Crit Care Med (2011) 183(12):1595–604. 10.1164/rccm.201011-1783OC 21471092

[5] Ogra PL Effect of tonsillectomy and adenoidectomy on nasopharyngeal antibody response to poliovirus. N Engl J Med (1971) 284(2):59–64. 10.1056/NEJM197101142840201 4321186

[6] Shikina T Hiroi T Iwatani K Jang MH Fukuyama S Tamura M IgA class switch occurs in the organized nasopharynx- and gut-associated lymphoid tissue, but not in the diffuse lamina propria of airways and gut. J Immunol (2004) 172(10):6259–64. 10.4049/jimmunol.172.10.6259 15128814

[7] Jahnsen FL Gran E Haye R Brandtzaeg P Human nasal mucosa contains antigen-presenting cells of strikingly different functional phenotypes. Am J Respir Cell Mol Biol (2004) 30(1):31–7. 10.1165/rcmb.2002-0230OC 12829449

[8] Kumar N Arthur CP Ciferri C Matsumoto ML Structure of the secretory immunoglobulin A core. Sci (New York NY) (2020) 367(6481):1008–14. 10.1126/science.aaz5807 32029686

[9] Muramatsu M Yoshida R Yokoyama A Miyamoto H Kajihara M Maruyama J Comparison of antiviral activity between IgA and IgG specific to influenza virus hemagglutinin: increased potential of IgA for heterosubtypic immunity. PLoS One (2014) 9(1):e85582. 10.1371/journal.pone.0085582 24465606 PMC3895000

[10] Yan H Lamm ME Björling E Huang YT Multiple functions of immunoglobulin A in mucosal defense against viruses: an in vitro measles virus model. J Virol (2002) 76(21):10972–9. 10.1128/jvi.76.21.10972-10979.2002 PMC136625 12368340

[11] Rogier EW Frantz AL Bruno ME Kaetzel CS Secretory IgA is Concentrated in the Outer Layer of Colonic Mucus along with Gut Bacteria. Pathogens (2014) 3(2):390–403. 10.3390/pathogens3020390 25437806 PMC4243452

[12] Marshall LJ Perks B Ferkol T Shute JK IL-8 released constitutively by primary bronchial epithelial cells in culture forms an inactive complex with secretory component. J Immunol (2001) 167(5):2816–23. 10.4049/jimmunol.167.5.2816 11509627

[13] Zuo Y Yalavarthi S Shi H Gockman K Zuo M Madison JA Neutrophil extracellular traps in COVID-19. JCI Insight (2020) 5(11):e138999. 10.1172/jci.insight.138999 PMC7308057 32329756

[14] Mazanec MB Kaetzel CS Lamm ME Fletcher D Nedrud JG Intracellular neutralization of virus by immunoglobulin A antibodies. Proc Natl Acad Sci U S A (1992) 89(15):6901–5. 10.1073/pnas.89.15.6901 PMC49612 1323121

[15] Csencsits KL Jutila MA Pascual DW Nasal-associated lymphoid tissue: phenotypic and functional evidence for the primary role of peripheral node addressin in naive lymphocyte adhesion to high endothelial venules in a mucosal site. J Immunol (Baltimore Md 1950) (1999) 163(3):1382–9. 10415038

[16] Csencsits KL Jutila MA Pascual DW Mucosal addressin expression and binding-interactions with naive lymphocytes vary among the cranial, oral, and nasal-associated lymphoid tissues. Eur J Immunol (2002) 32(11):3029–39. 10.1002/1521-4141(200211)32:11<3029::AID-IMMU3029>3.0.CO;2-9 12385022

[17] Brandtzaeg P Secretory immunity with special reference to the oral cavity. J Oral Microbiol (2013) 5. 10.3402/jom.v5i0.20401 PMC3595421 23487566

[18] Xu B Wagner N Pham LN Magno V Shan Z Butcher EC Lymphocyte homing to bronchus-associated lymphoid tissue (BALT) is mediated by L-selectin/PNAd, alpha4beta1 integrin/VCAM-1, and LFA-1 adhesion pathways. J Exp Med (2003) 197(10):1255–67. 10.1084/jem.20010685 PMC2193791 12756264

[19] VanCott TC Kaminski RW Mascola JR Kalyanaraman VS Wassef NM Alving CR HIV-1 neutralizing antibodies in the genital and respiratory tracts of mice intranasally immunized with oligomeric gp160. J Immunol (Baltimore Md 1950) (1998) 160(4):2000–12. 9469464

[20] Pizzolla A Wang Z Groom JR Kedzierska K Brooks AG Reading PC Nasal-associated lymphoid tissues (NALTs) support the recall but not priming of influenza virus-specific cytotoxic T cells. Proc Natl Acad Sci U S A (2017) 114(20):5225–30. 10.1073/pnas.1620194114 PMC5441821 28461487

[21] Channappanavar R Fett C Zhao J Meyerholz DK Perlman S Virus-specific memory CD8 T cells provide substantial protection from lethal severe acute respiratory syndrome coronavirus infection. J Virol (2014) 88(19):11034–44. 10.1128/JVI.01505-14 PMC4178831 25056892

[22] Cao X COVID-19: immunopathology and its implications for therapy. Nat Rev Immunol (2020) 20(5):269–70. 10.1038/s41577-020-0308-3 PMC7143200 32273594

[23] Jiang Y Wei X Guan J Qin S Wang Z Lu H COVID-19 pneumonia: CD8^+^ T and NK cells are decreased in number but compensatory increased in cytotoxic potential. Clin Immunol (Orlando Fla) (2020) 218:108516. 10.1016/j.clim.2020.108516 PMC7305921 32574709

[24] Lucas C Wong P Klein J Castro T Silva J Sundaram M Longitudinal analyses reveal immunological misfiring in severe COVID-19. Nature (2020) 584(7821):463–9. 10.1038/s41586-020-2588-y PMC7477538 32717743

[25] Sealy R Jones BG Surman SL Hurwitz JL Robust IgA and IgG-producing antibody forming cells in the diffuse-NALT and lungs of Sendai virus-vaccinated cotton rats associate with rapid protection against human parainfluenza virus-type 1. Vaccine (2010) 28(41):6749–56. 10.1016/j.vaccine.2010.07.068 PMC2950074 20682364

[26] Terauchi Y Sano K Ainai A Saito S Taga Y Ogawa-Goto K IgA polymerization contributes to efficient virus neutralization on human upper respiratory mucosa after intranasal inactivated influenza vaccine administration. Hum vaccines Immunother (2018) 14(6):1351–61. 10.1080/21645515.2018.1438791 PMC6037454 29425074

[27] Lowell GH Kaminski RW Grate S Hunt RE Charney C Zimmer S Intranasal and intramuscular proteosome-staphylococcal enterotoxin B (SEB) toxoid vaccines: immunogenicity and efficacy against lethal SEB intoxication in mice. Infect Immun (1996) 64(5):1706–13. 10.1128/IAI.64.5.1706-1713.1996 PMC173982 8613381

[28] Liu A Wang W Zhao X Zhou X Yang D Lu M Lv Y Disappearance of antibodies to SARS-CoV-2 in a -COVID-19 patient after recovery. Clin Microbiol Infect (2020) S1198–743X(20)30411-0. 10.1016/j.cmi.2020.07.009 PMC7346807 32653658

[29] Liang B Hyland L Hou S Nasal-associated lymphoid tissue is a site of long-term virus-specific antibody production following respiratory virus infection of mice. J Virol (2001) 75(11):5416–20. 10.1128/JVI.75.11.5416-5420.2001 PMC114951 11333927

[30] Walls AC Park YJ Tortorici MA Wall A McGuire AT Veesler D Structure, Function, and Antigenicity of the SARS-CoV-2 Spike Glycoprotein. Cell (2020) 181(2):281–292.e6. 10.1016/j.cell.2020.02.058 32155444 PMC7102599

[31] Hou YJ Okuda K Edwards CE Martinez DR Asakura T Dinnon KH SARS-CoV-2 Reverse Genetics Reveals a Variable Infection Gradient in the Respiratory Tract. Cell (2020) 182(2):426–46.e14. 10.1016/j.cell.2020.05.042 PMC7250779 32526206

[32] Sims AC Baric RS Yount B Burkett SE Collins PL Pickles RJ Severe acute respiratory syndrome coronavirus infection of human ciliated airway epithelia: role of ciliated cells in viral spread in the conducting airways of the lungs. J Virol (2005) 79(24):15511–24. 10.1128/JVI.79.24.15511-15524.2005 PMC1316022 16306622

[33] Liu L Wei Q Alvarez X Wang H Du Y Zhu H Epithelial cells lining salivary gland ducts are early target cells of severe acute respiratory syndrome coronavirus infection in the upper respiratory tracts of rhesus macaques. J Virol (2011) 85(8):4025–30. 10.1128/JVI.02292-10 PMC3126125 21289121

[34] Isho B Abe KT Zuo M Jamal AJ Rathod B Samavarchi-Tehrani P Mucosal versus systemic antibody responses to SARS-CoV-2 antigens in COVID-19 patients. medRxiv (2020). 10.1101/2020.08.01.20166553

[35] Yu HQ Sun BQ Fang ZF Zhao JC Liu XY Li YM Distinct features of SARS-CoV-2-specific IgA response in COVID-19 patients. Eur Respir J (2020) 56(2):2001526. 10.1183/13993003.01526-2020 32398307 PMC7236821

[36] Ma H Zeng W He H Zhao D Jiang D Zhou P Serum IgA, IgM, and IgG responses in COVID-19. Cell Mol Immunol (2020) 17(7):773–5. 10.1038/s41423-020-0474-z PMC7331804 32467617

[37] Varadhachary A Chatterjee D Garza J Garr RP Foley C Letkeman AF Salivary anti-SARS-CoV-2 IgA as an accessible biomarker of mucosal immunity against COVID-19. medRxiv preprint server Health Sci (2020). 10.1101/2020.08.07.20170258

[38] Liu L Wei Q Nishiura K Peng J Wang H Midkiff C Spatiotemporal interplay of severe acute respiratory syndrome coronavirus and respiratory mucosal cells drives viral dissemination in rhesus macaques. Mucosal Immunol (2016) 9(4):1089–101. 10.1038/mi.2015.127 PMC4900951 26647718

[39] Wölfel R Corman VM Guggemos W Seilmaier M Zange S Müller MA Virological assessment of hospitalized patients with COVID-2019. Nature (2020) 581(7809):465–9. 10.1038/s41586-020-2196-x 32235945

[40] Iwasaki A Yang Y The potential danger of suboptimal antibody responses in COVID-19. Nat Rev Immunol (2020) 20(6):339–41. 10.1038/s41577-020-0321-6 PMC7187142 32317716

[41] Sette A Crotty S Pre-existing immunity to SARS-CoV-2: the knowns and unknowns. Nat Rev Immunol (2020), 1–2. 10.1038/s41577-020-0389-z 32636479 PMC7339790

[42] Gorse GJ Patel GB Vitale JN O’Connor TZ Prevalence of antibodies to four human coronaviruses is lower in nasal secretions than in serum. Clin Vaccine Immunol CVI (2010) 17(12):1875–80. 10.1128/CVI.00278-10 PMC3008199 20943876

[43] Braun J Loyal L Frentsch M Wendisch D Georg P Kurth F SARS-CoV-2-reactive T cells in healthy donors and patients with COVID-19. Nature (2020). 10.1038/s41586-020-2598-9 32726801

[44] Eroshenko N Gill T Keaveney MK Church GM Trevejo JM Rajaniemi H Implications of antibody-dependent enhancement of infection for SARS-CoV-2 countermeasures. Nat Biotechnol (2020) 38(7):789–91. 10.1038/s41587-020-0577-1 32504046

[45] Mitchison NA T-cell-B-cell cooperation. Nat Rev Immunol (2004) 4(4):308–12. 10.1038/nri1334 15057789

[46] Taylor A Foo SS Bruzzone R Dinh LV King NJ Mahalingam S Fc receptors in antibody-dependent enhancement of viral infections. Immunol Rev (2015) 268(1):340–64. 10.1111/imr.12367 PMC7165974 26497532

[47] Pyzik M Sand K Hubbard JJ Andersen JT Sandlie I Blumberg RS The Neonatal Fc Receptor (FcRn): A Misnomer? Front Immunol (2019) 10:1540. 10.3389/fimmu.2019.01540 31354709 PMC6636548

[48] Smatti MK Al Thani AA Yassine HM Viral-Induced Enhanced Disease Illness. Front Microbiol (2018) 9:2991. 10.3389/fmicb.2018.02991 30568643 PMC6290032

[49] Jaume M Yip MS Cheung CY Leung HL Li PH Kien F Anti-severe acute respiratory syndrome coronavirus spike antibodies trigger infection of human immune cells via a pH- and cysteine protease-independent FcγR pathway. J Virol (2011) 85(20):10582–97. 10.1128/JVI.00671-11 PMC3187504 21775467

[50] Yip MS Leung NH Cheung CY Li PH Lee HH Daëron M Antibody-dependent infection of human macrophages by severe acute respiratory syndrome coronavirus. Virol J (2014) 11:82. 10.1186/1743-422X-11-82 24885320 PMC4018502

[51] Yasui F Kai C Kitabatake M Inoue S Yoneda M Yokochi S Prior immunization with severe acute respiratory syndrome (SARS)-associated coronavirus (SARS-CoV) nucleocapsid protein causes severe pneumonia in mice infected with SARS-CoV. J Immunol (2008) 181(9):6337–48. 10.4049/jimmunol.181.9.6337 18941225

[52] Bell TJ Brand OJ Morgan DJ Salek-Ardakani S Jagger C Fujimori Defective lung function following influenza virus is due to prolonged, reversible hyaluronan synthesis. Matrix Biol (2019) 80:14–28. 10.1016/j.matbio.2018.06.006 29933044 PMC6548309

[53] Janoff EN Wahl SM Thomas K Smith PD Modulation of human immunodeficiency virus type 1 infection of human monocytes by IgA. J Infect Dis (1995) 172(3):855–8. 10.1093/infdis/172.3.855 7658082

[54] Kozlowski PA Black KP Shen L Jackson S High prevalence of serum IgA HIV-1 infection-enhancing antibodies in HIV-infected persons. Masking by IgG. J Immunol (Baltimore Md 1950) (1995) 154(11):6163–73. 7751656

[55] Wan Y Shang J Sun S Tai W Chen J Geng Q Molecular Mechanism for Antibody-Dependent Enhancement of Coronavirus Entry. J Virol (2020) 94(5):e02015–19. 10.1128/JVI.02015-19 PMC7022351 31826992

[56] Liu L Wei Q Lin Q Fang J Wang H Kwo H Anti-spike IgG causes severe acute lung injury by skewing macrophage responses during acute SARS-CoV infection. JCI Insight (2019) 4(4):e123158. 10.1172/jci.insight.123158 PMC6478436 30830861

[57] Latvala S Jacobsen B Otteneder MB Herrmann A Kronenberg S Distribution of FcRn Across Species and Tissues. J Histochem Cytochem (2017) 65(6):321–33. 10.1369/0022155417705095 PMC5625855 28402755

[58] Tzaban S Massol RH Yen E Hamman W Frank SR Lapierre LA The recycling and transcytotic pathways for IgG transport by FcRn are distinct and display an inherent polarity. J Cell Biol (2009) 185(4):673–84. 10.1083/jcb.200809122 PMC2711563 19451275

[59] Ward ES Martinez C Vaccaro C Zhou J Tang Q Ober RJ From sorting endosomes to exocytosis: association of Rab4 and Rab11 GTPases with the Fc receptor, FcRn, during recycling. Mol Biol Cell (2005) 16(4):2028–38. 10.1091/mbc.e04-08-0735 PMC1073680 15689494

[60] Burkard C Verheije MH Wicht O van Kasteren S II van Kuppeveld FJ Haagmans BL Coronavirus cell entry occurs through the endo-/lysosomal pathway in a proteolysis-dependent manner. PLoS Pathog (2014) 10(11):e1004502. 10.1371/journal.ppat.1004502 25375324 PMC4223067

[61] Labò N Ohnuki H Tosato G Vasculopathy and Coagulopathy Associated with SARS-CoV-2 Infection. Cells (2020) 9(7):1583. 10.3390/cells9071583 PMC7408139 32629875

[62] Moreira A Kawasaki disease linked to COVID-19 in children. Nat Rev Immunol (2020) 20(7):407. 10.1038/s41577-020-0350-1 PMC7251555 32461672

[63] Su F Patel GB Hu S Chen W Induction of mucosal immunity through systemic immunization: Phantom or reality? Hum Vaccines Immunother (2016) 12(4):1070–9. 10.1080/21645515.2015.1114195 PMC4962944 26752023

[64] Clements JD Freytag LC Parenteral Vaccination Can Be an Effective Means of Inducing Protective Mucosal Responses. Clin Vaccine Immunol CVI (2016) 23(6):438–41. 10.1128/CVI.00214-16 PMC4895008 27122485

[65] Russell MW Sibley DA Nikolova EB Tomana M Mestecky J IgA antibody as a non-inflammatory regulator of immunity. Biochem Soc Trans (1997) 25(2):466–70. 10.1042/bst0250466 9191137

[66] Nikolova EB Tomana M Russell MW All forms of human IgA antibodies bound to antigen interfere with complement (C3) fixation induced by IgG or by antigen alone. Scand J Immunol (1994) 39(3):275–80. 10.1111/j.1365-3083.1994.tb03371.x 8128187

[67] Mestecky J Russell MW Elson CO Perspectives on mucosal vaccines: is mucosal tolerance a barrier? J Immunol (2007) 179(9):5633–8. 10.4049/jimmunol.179.9.5633 17947632

[68] Yap JKY Moriyama M Iwasaki A Inflammasomes and Pyroptosis as Therapeutic Targets for COVID-19. J Immunol (2020) 205(2):307–12. 10.4049/jimmunol.2000513 PMC7343621 32493814

[69] Du L Zhao G Lin Y Sui H Chan C Ma S Intranasal vaccination of recombinant adeno-associated virus encoding receptor-binding domain of severe acute respiratory syndrome coronavirus (SARS-CoV) spike protein induces strong mucosal immune responses and provides long-term protection against SARS-CoV infection. J Immunol (2008) 180(2):948–56. 10.4049/jimmunol.180.2.948 PMC2603051 18178835

[70] Kim MH Kim HJ Chang J Superior immune responses induced by intranasal immunization with recombinant adenovirus-based vaccine expressing full-length Spike protein of Middle East respiratory syndrome coronavirus. PLoS One (2019) 14(7):e0220196. 10.1371/journal.pone.0220196 31329652 PMC6645677

[71] Hassan AO Case JB Winkler ES Thackray LB Kafai NM Bailey AL A SARS-CoV-2 Infection Model in Mice Demonstrates Protection by Neutralizing Antibodies. Cell (2020) 182(3):744–53.e4. 10.1016/j.cell.2020.06.011 32553273 PMC7284254

[72] Fujihashi K Sato S Kiyono H Mucosal adjuvants for vaccines to control upper respiratory infections in the elderly. Exp Gerontol (2014) 54:21–6. 10.1016/j.exger.2014.01.006 PMC3989367 24440991

[73] Sasaki E Asanuma H Momose H Furuhata K Mizukami T Hamaguchi I Immunogenicity and Toxicity of Different Adjuvants Can Be Characterized by Profiling Lung Biomarker Genes After Nasal Immunization. Front Immunol (2020) 11(2171). 10.3389/fimmu.2020.02171 PMC7516075 33013912

[74] Hotez PJ Bottazzi ME Corry DB The potential role of Th17 immune responses in coronavirus immunopathology and vaccine-induced immune enhancement. Microbes Infect (2020) 22(4-5):165–7. 10.1016/j.micinf.2020.04.005 PMC7162764 32305501

[75] Mitragotri S Immunization without needles. Nat Rev Immunol (2005) 5(12):905–16. 10.1038/nri1728 16239901

[76] World Health Organization State of the World’s Vaccines and Immunization. Geneva: World Health Organization (1996). Available at: https://apps.who.int/iris/bitstream/handle/10665/44169/9789241563864_eng.pdf?sequence=1.

